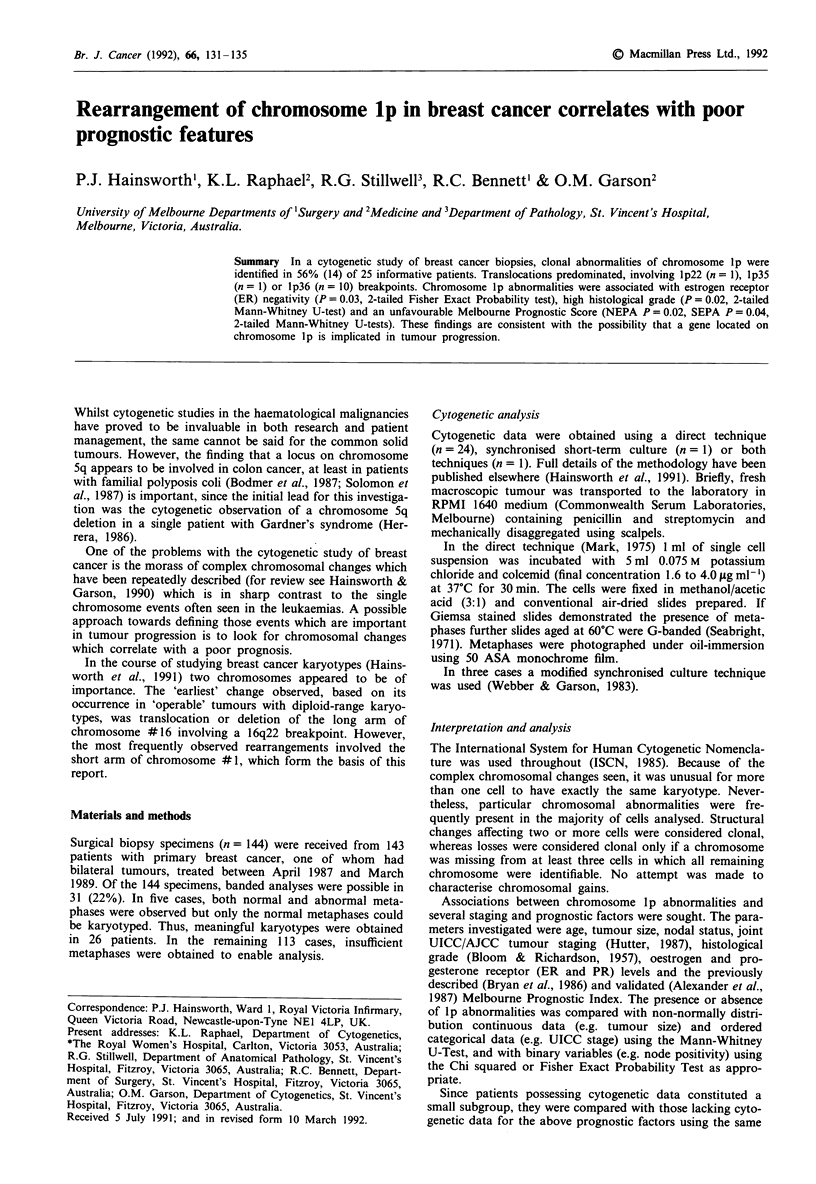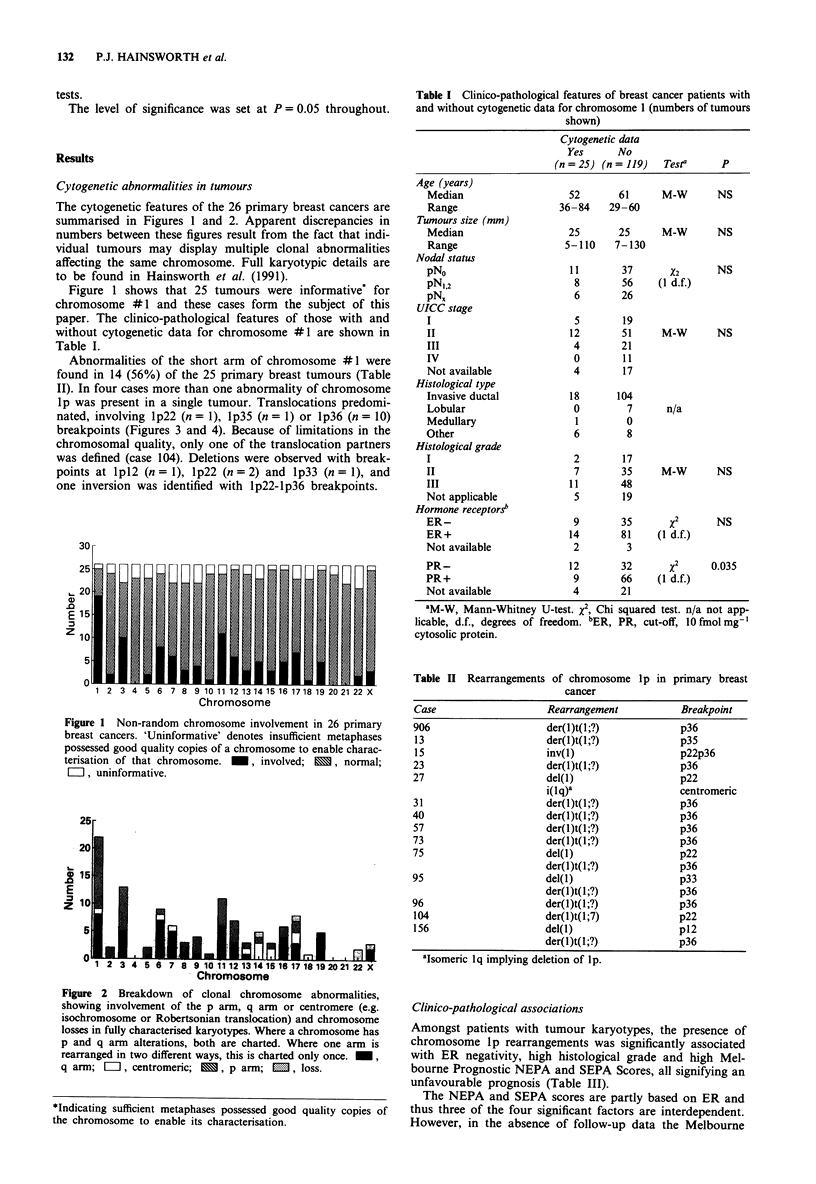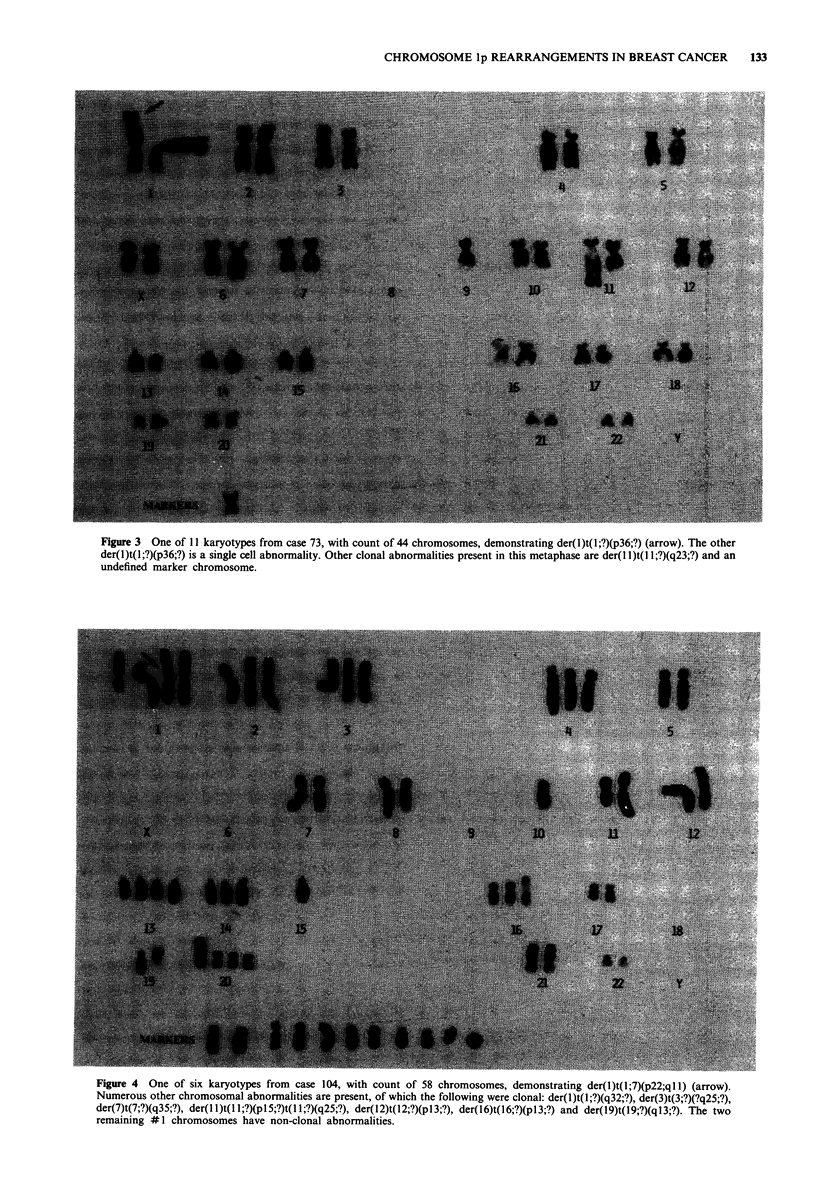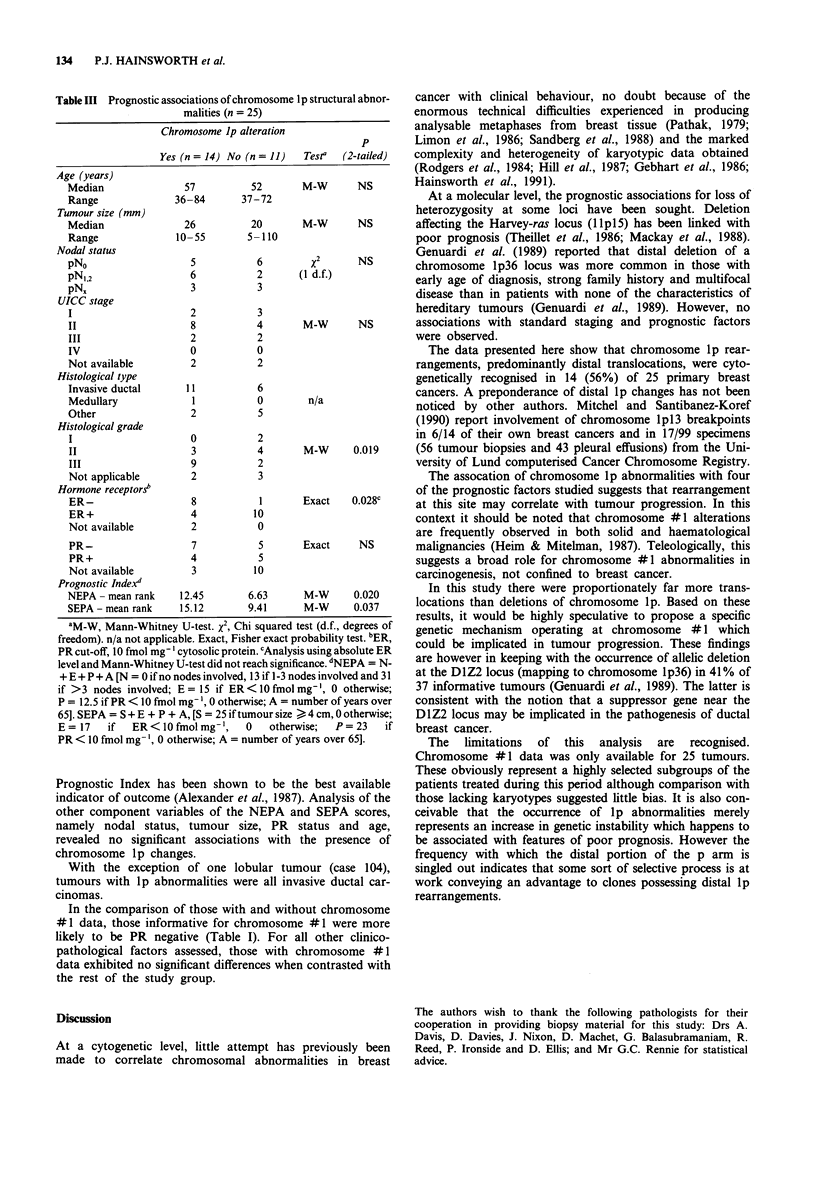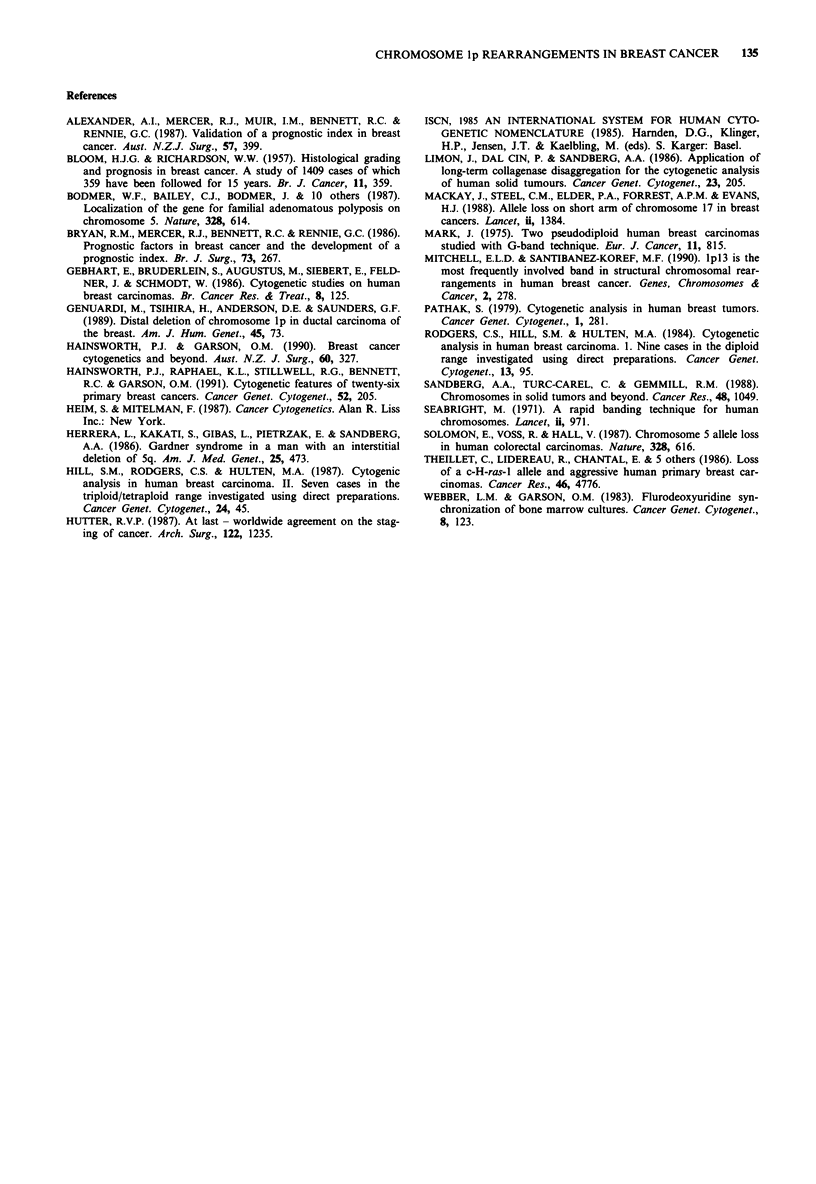# Rearrangement of chromosome 1p in breast cancer correlates with poor prognostic features.

**DOI:** 10.1038/bjc.1992.229

**Published:** 1992-07

**Authors:** P. J. Hainsworth, K. L. Raphael, R. G. Stillwell, R. C. Bennett, O. M. Garson

**Affiliations:** University of Melbourne Department of Surgery, St. Vincent's Hospital, Victoria, Australia.

## Abstract

**Images:**


*Br. J. Cancer* (1992), **66**, 131–135 © Macmillan Press Ltd., 1992

### Rearrangement of chromosome 1p in breast cancer correlates with poor prognostic features

P.J. Hainsworth[1], K.L. Raphael[2], R.G. Stillwell[3], R.C. Bennett[1] & O.M. Garson[2]

*University of Melbourne Departments of [1]Surgery and [2]Medicine and [3]Department of Pathology, St. Vincent's Hospital, Melbourne, Victoria, Australia.*

**Summary** In a cytogenetic study of breast cancer biopsies, clonal abnormalities of chromosome 1p were identified in 56% (14) of 25 informative patients. Translocations predominated, involving 1p22 ($n = 1$), 1p35 ($n = 1$) or 1p36 ($n = 10$) breakpoints. Chromosome 1p abnormalities were associated with estrogen receptor (ER) negativity ($P = 0.03$, 2-tailed Fisher Exact Probability test), high histological grade ($P = 0.02$, 2-tailed Mann-Whitney U-test) and an unfavourable Melbourne Prognostic Score (NEPA $P = 0.02$, SEPA $P = 0.04$, 2-tailed Mann-Whitney U-tests). These findings are consistent with the possibility that a gene located on chromosome 1p is implicated in tumour progression.

Whilst cytogenetic studies in the haematological malignancies have proved to be invaluable in both research and patient management, the same cannot be said for the common solid tumours. However, the finding that a locus on chromosome 5q appears to be involved in colon cancer, at least in patients with familial polyposis coli (Bodmer *et al*., 1987; Solomon *et al*., 1987) is important, since the initial lead for this investigation was the cytogenetic observation of a chromosome 5q deletion in a single patient with Gardner's syndrome (Herrera, 1986).

One of the problems with the cytogenetic study of breast cancer is the morass of complex chromosomal changes which have been repeatedly described (for review see Hainsworth & Garson, 1990) which is in sharp contrast to the single chromosome events often seen in the leukaemias. A possible approach towards defining those events which are important in tumour progression is to look for chromosomal changes which correlate with a poor prognosis.

In the course of studying breast cancer karyotypes (Hainsworth *et al*., 1991) two chromosomes appeared to be of importance. The 'earliest' change observed, based on its occurrence in 'operable' tumours with diploid-range karyotypes, was translocation or deletion of the long arm of chromosome #16 involving a 16q22 breakpoint. However, the most frequently observed rearrangements involved the short arm of chromosome #1, which form the basis of this report.

### Materials and methods

Surgical biopsy specimens ($n = 144$) were received from 143 patients with primary breast cancer, one of whom had bilateral tumours, treated between April 1987 and March 1989. Of the 144 specimens, banded analyses were possible in 31 (22%). In five cases, both normal and abnormal metaphases were observed but only the normal metaphases could be karyotyped. Thus, meaningful karyotypes were obtained in 26 patients. In the remaining 113 cases, insufficient metaphases were obtained to enable analysis.

Correspondence: P.J. Hainsworth, Ward 1, Royal Victoria Infirmary, Queen Victoria Road, Newcastle-upon-Tyne NE1 4LP, UK.
Present addresses: K.L. Raphael, Department of Cytogenetics, *The Royal Women's Hospital, Carlton, Victoria 3053, Australia; R.G. Stillwell, Department of Anatomical Pathology, St. Vincent's Hospital, Fitzroy, Victoria 3065, Australia; R.C. Bennett, Department of Surgery, St. Vincent's Hospital, Fitzroy, Victoria 3065, Australia; O.M. Garson, Department of Cytogenetics, St. Vincent's Hospital, Fitzroy, Victoria 3065, Australia.
Received 5 July 1991; and in revised form 10 March 1992.

### *Cytogenetic analysis*

Cytogenetic data were obtained using a direct technique ($n = 24$), synchronised short-term culture ($n = 1$) or both techniques ($n = 1$). Full details of the methodology have been published elsewhere (Hainsworth *et al*., 1991). Briefly, fresh macroscopic tumour was transported to the laboratory in RPMI 1640 medium (Commonwealth Serum Laboratories, Melbourne) containing penicillin and streptomycin and mechanically disaggregated using scalpels.

In the direct technique (Mark, 1975) 1 ml of single cell suspension was incubated with 5 ml 0.075 M potassium chloride and colcemid (final concentration 1.6 to 4.0 $\mu g\ ml^{-1}$) at 37°C for 30 min. The cells were fixed in methanol/acetic acid (3:1) and conventional air-dried slides prepared. If Giemsa stained slides demonstrated the presence of metaphases further slides aged at 60°C were G-banded (Seabright, 1971). Metaphases were photographed under oil-immersion using 50 ASA monochrome film.

In three cases a modified synchronised culture technique was used (Webber & Garson, 1983).

### *Interpretation and analysis*

The International System for Human Cytogenetic Nomenclature was used throughout (ISCN, 1985). Because of the complex chromosomal changes seen, it was unusual for more than one cell to have exactly the same karyotype. Nevertheless, particular chromosomal abnormalities were frequently present in the majority of cells analysed. Structural changes affecting two or more cells were considered clonal, whereas losses were considered clonal only if a chromosome was missing from at least three cells in which all remaining chromosome were identifiable. No attempt was made to characterise chromosomal gains.

Associations between chromosome 1p abnormalities and several staging and prognostic factors were sought. The parameters investigated were age, tumour size, nodal status, joint UICC/AJCC tumour staging (Hutter, 1987), histological grade (Bloom & Richardson, 1957), oestrogen and progesterone receptor (ER and PR) levels and the previously described (Bryan *et al*., 1986) and validated (Alexander *et al*., 1987) Melbourne Prognostic Index. The presence or absence of 1p abnormalities was compared with non-normally distribution continuous data (e.g. tumour size) and ordered categorical data (e.g. UICC stage) using the Mann-Whitney U-Test, and with binary variables (e.g. node positivity) using the Chi squared or Fisher Exact Probability Test as appropriate.

Since patients possessing cytogenetic data constituted a small subgroup, they were compared with those lacking cytogenetic data for the above prognostic factors using the same tests.

The level of significance was set at $P = 0.05$ throughout.

### Results

### *Cytogenetic abnormalities in tumours*

The cytogenetic features of the 26 primary breast cancers are summarised in Figures 1 and 2. Apparent discrepancies in numbers between these figures result from the fact that individual tumours may display multiple clonal abnormalities affecting the same chromosome. Full karyotypic details are to be found in Hainsworth *et al.* (1991).

Figure 1 shows that 25 tumours were informative* for chromosome #1 and these cases form the subject of this paper. The clinico-pathological features of those with and without cytogenetic data for chromosome #1 are shown in Table I.

Abnormalities of the short arm of chromosome #1 were found in 14 (56%) of the 25 primary breast tumours (Table II). In four cases more than one abnormality of chromosome 1p was present in a single tumour. Translocations predominated, involving 1p22 ($n = 1$), 1p35 ($n = 1$) or 1p36 ($n = 10$) breakpoints (Figures 3 and 4). Because of limitations in the chromosomal quality, only one of the translocation partners was defined (case 104). Deletions were observed with breakpoints at 1p12 ($n = 1$), 1p22 ($n = 2$) and 1p33 ($n = 1$), and one inversion was identified with 1p22-1p36 breakpoints.

**Figure 1** Non-random chromosome involvement in 26 primary breast cancers. 'Uninformative' denotes insufficient metaphases possessed good quality copies of a chromosome to enable characterisation of that chromosome. ■, involved; ▨, normal; □, uninformative.

**Figure 2** Breakdown of clonal chromosome abnormalities, showing involvement of the p arm, q arm or centromere (e.g. isochromosome or Robertsonian translocation) and chromosome losses in fully characterised karyotypes. Where a chromosome has p and q arm alterations, both are charted. Where one arm is rearranged in two different ways, this is charted only once. ■, q arm; □, centromeric; ▨, p arm; ▩, loss.

*Indicating sufficient metaphases possessed good quality copies of the chromosome to enable its characterisation.

**Table I** Clinico-pathological features of breast cancer patients with and without cytogenetic data for chromosome 1 (numbers of tumours shown)

| | *Cytogenetic data* *Yes* *(n = 25)* | *No* *(n = 119)* | *Test*[a] | *P* |
|---|---|---|---|---|
| *Age (years)* | | | | |
| Median | 52 | 61 | M-W | NS |
| Range | 36–84 | 29–60 | | |
| *Tumours size (mm)* | | | | |
| Median | 25 | 25 | M-W | NS |
| Range | 5–110 | 7–130 | | |
| *Nodal status* | | | | |
| $pN_0$ | 11 | 37 | $\chi_2$ | NS |
| $pN_{1,2}$ | 8 | 56 | (1 d.f.) | |
| $pN_x$ | 6 | 26 | | |
| *UICC stage* | | | | |
| I | 5 | 19 | | |
| II | 12 | 51 | M-W | NS |
| III | 4 | 21 | | |
| IV | 0 | 11 | | |
| Not available | 4 | 17 | | |
| *Histological type* | | | | |
| Invasive ductal | 18 | 104 | | |
| Lobular | 0 | 7 | n/a | |
| Medullary | 1 | 0 | | |
| Other | 6 | 8 | | |
| *Histological grade* | | | | |
| I | 2 | 17 | | |
| II | 7 | 35 | M-W | NS |
| III | 11 | 48 | | |
| Not applicable | 5 | 19 | | |
| *Hormone receptors*[b] | | | | |
| ER− | 9 | 35 | $\chi^2$ | NS |
| ER+ | 14 | 81 | (1 d.f.) | |
| Not available | 2 | 3 | | |
| PR− | 12 | 32 | $\chi^2$ | 0.035 |
| PR+ | 9 | 66 | (1 d.f.) | |
| Not available | 4 | 21 | | |

[a]M-W, Mann-Whitney U-test. $\chi^2$, Chi squared test. n/a not applicable, d.f., degrees of freedom. [b]ER, PR, cut-off, 10 fmol $mg^{-1}$ cytosolic protein.

**Table II** Rearrangements of chromosome 1p in primary breast cancer

| *Case* | *Rearrangement* | *Breakpoint* |
|---|---|---|
| 906 | der(1)t(1;?) | p36 |
| 13 | der(1)t(1;?) | p35 |
| 15 | inv(1) | p22p36 |
| 23 | der(1)t(1;?) | p36 |
| 27 | del(1) | p22 |
| | i(1q)[a] | centromeric |
| 31 | der(1)t(1;?) | p36 |
| 40 | der(1)t(1;?) | p36 |
| 57 | der(1)t(1;?) | p36 |
| 73 | der(1)t(1;?) | p36 |
| 75 | del(1) | p22 |
| | der(1)t(1;?) | p36 |
| 95 | del(1) | p33 |
| | der(1)t(1;?) | p36 |
| 96 | der(1)t(1;?) | p36 |
| 104 | der(1)t(1;7) | p22 |
| 156 | del(1) | p12 |
| | der(1)t(1;?) | p36 |

[a]Isomeric 1q implying deletion of 1p.

### *Clinico-pathological associations*

Amongst patients with tumour karyotypes, the presence of chromosome 1p rearrangements was significantly associated with ER negativity, high histological grade and high Melbourne Prognostic NEPA and SEPA Scores, all signifying an unfavourable prognosis (Table III).

The NEPA and SEPA scores are partly based on ER and thus three of the four significant factors are interdependent. However, in the absence of follow-up data the Melbourne

**Figure 3** One of 11 karyotypes from case 73, with count of 44 chromosomes, demonstrating der(1)t(1;?)(p36;?) (arrow). The other der(1)t(1;?)(p36;?) is a single cell abnormality. Other clonal abnormalities present in this metaphase are der(11)t(11;?)(q23;?) and an undefined marker chromosome.

**Figure 4** One of six karyotypes from case 104, with count of 58 chromosomes, demonstrating der(1)t(1;7)(p22;q11) (arrow). Numerous other chromosomal abnormalities are present, of which the following were clonal: der(1)t(1;?)(q32;?), der(3)t(3;?)(?q25;?), der(7)t(7;?)(q35;?), der(11)t(11;?)(p15;?)t(11;?)(q25;?), der(12)t(12;?)(p13;?), der(16)t(16;?)(p13;?) and der(19)t(19;?)(q13;?). The two remaining #1 chromosomes have non-clonal abnormalities.

**Table III** Prognostic associations of chromosome 1p structural abnormalities ($n = 25$)

| | *Chromosome 1p alteration* | | | *P* |
|---|---|---|---|---|
| | *Yes (n = 14)* | *No (n = 11)* | *Test*[a] | *(2-tailed)* |
| *Age (years)* | | | | |
| Median | 57 | 52 | M-W | NS |
| Range | 36–84 | 37–72 | | |
| *Tumour size (mm)* | | | | |
| Median | 26 | 20 | M-W | NS |
| Range | 10–55 | 5–110 | | |
| *Nodal status* | | | | |
| $pN_0$ | 5 | 6 | $\chi^2$ | NS |
| $pN_{1,2}$ | 6 | 2 | (1 d.f.) | |
| $pN_x$ | 3 | 3 | | |
| *UICC stage* | | | | |
| I | 2 | 3 | | |
| II | 8 | 4 | M-W | NS |
| III | 2 | 2 | | |
| IV | 0 | 0 | | |
| Not available | 2 | 2 | | |
| *Histological type* | | | | |
| Invasive ductal | 11 | 6 | | |
| Medullary | 1 | 0 | n/a | |
| Other | 2 | 5 | | |
| *Histological grade* | | | | |
| I | 0 | 2 | | |
| II | 3 | 4 | M-W | 0.019 |
| III | 9 | 2 | | |
| Not applicable | 2 | 3 | | |
| *Hormone receptors*[b] | | | | |
| ER– | 8 | 1 | Exact | 0.028[c] |
| ER+ | 4 | 10 | | |
| Not available | 2 | 0 | | |
| PR– | 7 | 5 | Exact | NS |
| PR+ | 4 | 5 | | |
| Not available | 3 | 10 | | |
| *Prognostic Index*[d] | | | | |
| NEPA - mean rank | 12.45 | 6.63 | M-W | 0.020 |
| SEPA - mean rank | 15.12 | 9.41 | M-W | 0.037 |

[a]M-W, Mann-Whitney U-test. $\chi^2$, Chi squared test (d.f., degrees of freedom). n/a not applicable. Exact, Fisher exact probability test. [b]ER, PR cut-off, 10 fmol $mg^{-1}$ cytosolic protein. [c]Analysis using absolute ER level and Mann-Whitney U-test did not reach significance. [d]NEPA = N + E + P + A [N = 0 if no nodes involved, 13 if 1-3 nodes involved and 31 if >3 nodes involved; E = 15 if ER < 10 fmol $mg^{-1}$, 0 otherwise; P = 12.5 if PR < 10 fmol $mg^{-1}$, 0 otherwise; A = number of years over 65]. SEPA = S + E + P + A, [S = 25 if tumour size ⩾ 4 cm, 0 otherwise; E = 17 if ER < 10 fmol $mg^{-1}$, 0 otherwise; $P = 23$ if PR < 10 fmol $mg^{-1}$, 0 otherwise; A = number of years over 65].

Prognostic Index has been shown to be the best available indicator of outcome (Alexander *et al.*, 1987). Analysis of the other component variables of the NEPA and SEPA scores, namely nodal status, tumour size, PR status and age, revealed no significant associations with the presence of chromosome 1p changes.

With the exception of one lobular tumour (case 104), tumours with 1p abnormalities were all invasive ductal carcinomas.

In the comparison of those with and without chromosome #1 data, those informative for chromosome #1 were more likely to be PR negative (Table I). For all other clinicopathological factors assessed, those with chromosome #1 data exhibited no significant differences when contrasted with the rest of the study group.

### Discussion

At a cytogenetic level, little attempt has previously been made to correlate chromosomal abnormalities in breast cancer with clinical behaviour, no doubt because of the enormous technical difficulties experienced in producing analysable metaphases from breast tissue (Pathak, 1979; Limon *et al.*, 1986; Sandberg *et al.*, 1988) and the marked complexity and heterogeneity of karyotypic data obtained (Rodgers *et al.*, 1984; Hill *et al.*, 1987; Gebhart *et al.*, 1986; Hainsworth *et al.*, 1991).

At a molecular level, the prognostic associations for loss of heterozygosity at some loci have been sought. Deletion affecting the Harvey-*ras* locus (11p15) has been linked with poor prognosis (Theillet *et al.*, 1986; Mackay *et al.*, 1988). Genuardi *et al.* (1989) reported that distal deletion of a chromosome 1p36 locus was more common in those with early age of diagnosis, strong family history and multifocal disease than in patients with none of the characteristics of hereditary tumours (Genuardi *et al.*, 1989). However, no associations with standard staging and prognostic factors were observed.

The data presented here show that chromosome 1p rearrangements, predominantly distal translocations, were cytogenetically recognised in 14 (56%) of 25 primary breast cancers. A preponderance of distal 1p changes has not been noticed by other authors. Mitchel and Santibanez-Koref (1990) report involvement of chromosome 1p13 breakpoints in 6/14 of their own breast cancers and in 17/99 specimens (56 tumour biopsies and 43 pleural effusions) from the University of Lund computerised Cancer Chromosome Registry.

The assocation of chromosome 1p abnormalities with four of the prognostic factors studied suggests that rearrangement at this site may correlate with tumour progression. In this context it should be noted that chromosome #1 alterations are frequently observed in both solid and haematological malignancies (Heim & Mitelman, 1987). Teleologically, this suggests a broad role for chromosome #1 abnormalities in carcinogenesis, not confined to breast cancer.

In this study there were proportionately far more translocations than deletions of chromosome 1p. Based on these results, it would be highly speculative to propose a specific genetic mechanism operating at chromosome #1 which could be implicated in tumour progression. These findings are however in keeping with the occurrence of allelic deletion at the D1Z2 locus (mapping to chromosome 1p36) in 41% of 37 informative tumours (Genuardi *et al.*, 1989). The latter is consistent with the notion that a suppressor gene near the D1Z2 locus may be implicated in the pathogenesis of ductal breast cancer.

The limitations of this analysis are recognised. Chromosome #1 data was only available for 25 tumours. These obviously represent a highly selected subgroups of the patients treated during this period although comparison with those lacking karyotypes suggested little bias. It is also conceivable that the occurrence of 1p abnormalities merely represents an increase in genetic instability which happens to be associated with features of poor prognosis. However the frequency with which the distal portion of the p arm is singled out indicates that some sort of selective process is at work conveying an advantage to clones possessing distal 1p rearrangements.

The authors wish to thank the following pathologists for their cooperation in providing biopsy material for this study: Drs A. Davis, D. Davies, J. Nixon, D. Machet, G. Balasubramaniam, R. Reed, P. Ironside and D. Ellis; and Mr G.C. Rennie for statistical advice.

### References

ALEXANDER, A.I., MERCER, R.J., MUIR, I.M., BENNETT, R.C. & RENNIE, G.C. (1987). Validation of a prognostic index in breast cancer. *Aust. N.Z.J. Surg.*, **57**, 399.

BLOOM, H.J.G. & RICHARDSON, W.W. (1957). Histological grading and prognosis in breast cancer. A study of 1409 cases of which 359 have been followed for 15 years. *Br. J. Cancer*, **11**, 359.

BODMER, W.F., BAILEY, C.J., BODMER, J. & 10 others (1987). Localization of the gene for familial adenomatous polyposis on chromosome 5. *Nature*, **328**, 614.

BRYAN, R.M., MERCER, R.J., BENNETT, R.C. & RENNIE, G.C. (1986). Prognostic factors in breast cancer and the development of a prognostic index. *Br. J. Surg.*, **73**, 267.

GEBHART, E., BRUDERLEIN, S., AUGUSTUS, M., SIEBERT, E., FELDNER, J. & SCHMODT, W. (1986). Cytogenetic studies on human breast carcinomas. *Br. Cancer Res. & Treat.*, **8**, 125.

GENUARDI, M., TSIHIRA, H., ANDERSON, D.E. & SAUNDERS, G.F. (1989). Distal deletion of chromosome 1p in ductal carcinoma of the breast. *Am. J. Hum. Genet.*, **45**, 73.

HAINSWORTH, P.J. & GARSON, O.M. (1990). Breast cancer cytogenetics and beyond. *Aust. N.Z. J. Surg.*, **60**, 327.

HAINSWORTH, P.J., RAPHAEL, K.L., STILLWELL, R.G., BENNETT, R.C. & GARSON, O.M. (1991). Cytogenetic features of twenty-six primary breast cancers. *Cancer Genet. Cytogenet.*, **52**, 205.

HEIM, S. & MITELMAN, F. (1987). *Cancer Cytogenetics*. Alan R. Liss Inc.: New York.

HERRERA, L., KAKATI, S., GIBAS, L., PIETRZAK, E. & SANDBERG, A.A. (1986). Gardner syndrome in a man with an interstitial deletion of 5q. *Am. J. Med. Genet.*, **25**, 473.

HILL, S.M., RODGERS, C.S. & HULTEN, M.A. (1987). Cytogenic analysis in human breast carcinoma. II. Seven cases in the triploid/tetraploid range investigated using direct preparations. *Cancer Genet. Cytogenet.*, **24**, 45.

HUTTER, R.V.P. (1987). At last – worldwide agreement on the staging of cancer. *Arch. Surg.*, **122**, 1235.

ISCN, 1985 AN INTERNATIONAL SYSTEM FOR HUMAN CYTOGENETIC NOMENCLATURE (1985). Harnden, D.G., Klinger, H.P., Jensen, J.T. & Kaelbling, M. (eds). S. Karger: Basel.

LIMON, J., DAL CIN, P. & SANDBERG, A.A. (1986). Application of long-term collagenase disaggregation for the cytogenetic analysis of human solid tumours. *Cancer Genet. Cytogenet.*, **23**, 205.

MACKAY, J., STEEL, C.M., ELDER, P.A., FORREST, A.P.M. & EVANS, H.J. (1988). Allele loss on short arm of chromosome 17 in breast cancers. *Lancet*, **ii**, 1384.

MARK, J. (1975). Two pseudodiploid human breast carcinomas studied with G-band technique. *Eur. J. Cancer*, **11**, 815.

MITCHELL, E.L.D. & SANTIBANEZ-KOREF, M.F. (1990). 1p13 is the most frequently involved band in structural chromosomal rearrangements in human breast cancer. *Genes, Chromosomes & Cancer*, **2**, 278.

PATHAK, S. (1979). Cytogenetic analysis in human breast tumors. *Cancer Genet. Cytogenet.*, **1**, 281.

RODGERS, C.S., HILL, S.M. & HULTEN, M.A. (1984). Cytogenetic analysis in human breast carcinoma. 1. Nine cases in the diploid range investigated using direct preparations. *Cancer Genet. Cytogenet.*, **13**, 95.

SANDBERG, A.A., TURC-CAREL, C. & GEMMILL, R.M. (1988). Chromosomes in solid tumors and beyond. *Cancer Res.*, **48**, 1049.

SEABRIGHT, M. (1971). A rapid banding technique for human chromosomes. *Lancet*, **ii**, 971.

SOLOMON, E., VOSS, R. & HALL, V. (1987). Chromosome 5 allele loss in human colorectal carcinomas. *Nature*, **328**, 616.

THEILLET, C., LIDEREAU, R., CHANTAL, E. & 5 others (1986). Loss of a c-H-*ras*-1 allele and aggressive human primary breast carcinomas. *Cancer Res.*, **46**, 4776.

WEBBER, L.M. & GARSON, O.M. (1983). Flurodeoxyuridine synchronization of bone marrow cultures. *Cancer Genet. Cytogenet.*, **8**, 123.